# Metagenomic Insight of a Full Scale Eco-Friendly Treatment System of Textile Dye Wastewater Using Bioaugmentation of the Composite Culture CES-1

**DOI:** 10.3390/microorganisms9071503

**Published:** 2021-07-14

**Authors:** Aalfin Emmanuel Santhanarajan, Woo-Jun Sul, Keun-Je Yoo, Hoon-Je Seong, Hong-Gi Kim, Sung-Cheol Koh

**Affiliations:** 1 Division of Civil, Environmental Engineering and Logistics System, Korea Maritime and Ocean University, Busan 49112, Korea; eaalfi@gmail.com (A.E.S.); kjyoo@kmou.ac.kr (K.-J.Y.); 2 Department of Systems Biotechnology, Chung-Ang University, Anseong 06974, Korea; sulwj@cau.ac.kr (W.-J.S.); hoonjeseong@gmail.com (H.-J.S.); 3 Bayo Inc., Jinju 52665, Korea; hg-kim62@hanmail.net

**Keywords:** textile dye wastewater, bioaugmentation, sludge reduction, microbial diversity, metagenomic analysis

## Abstract

Effects of bioaugmentation of the composite microbial culture CES-1 on a full scale textile dye wastewater treatment process were investigated in terms of water quality, sludge reduction, dynamics of microbial community structures and their functional genes responsible for degradation of azo dye, and other chemicals. The removal efficiencies for Chemical Oxygen Demand (COD), Total Nitrogen (T-N), Total Phosphorus (T-P), Suspended Solids (SS), and color intensity (96.4%, 78.4, 83.1, 84.4, and 92.0, respectively) 300–531 days after the augmentation were generally improved after bioaugmentation. The denitrification linked to T-N removal appeared to contribute to the concomitant COD removal that triggered a reduction of sludge (up to 22%) in the same period of augmentation. Azo dye and aromatic compound degradation and other downstream pathways were highly metabolically interrelated. Augmentation of CES-1 increased microbial diversity in the later stages of augmentation when a strong microbial community selection of *Acinetobacter* *parvus*, *Acinetobacter* *johnsonii*, *Marinobacter manganoxydans*, *Verminephrobacter* sp., and *Arcobacter* sp. occurred. Herein, there might be a possibility that the CES-1 augmentation could facilitate the indigenous microbial community successions so that the selected communities made the augmentation successful. The metagenomic analysis turned out to be a reasonable and powerful tool to provide with new insights and useful biomarkers for the complex environmental conditions, such as the full scale dye wastewater treatment system undergoing bioaugmentation.

## 1. Introduction

Azo dyes are one of the oldest synthetic chemicals and are still widely used in textile printing and the food industries. The annual production worldwide is approximately 700,000 tons, about 10%–15% of which are released into the environment during manufacturing and usage [1]. The textile industry is considered one of the largest water consumers in the world. It is rapidly expanding, and dyes are also continuously being upgraded and replaced by superior compounds that have enhanced fastness, stability, brightness, and resistance to natural degradation. Color is the first contaminant to be recognized in the wastewater and has to be removed before discharging into water bodies or on land [2]. The presence of very small amounts of dyes in water (less than 1 ppm for some dyes) is highly visible and affects the aesthetic merit, water transparency, and gas solubility in lakes, rivers, and other water bodies [3]. It is noteworthy that some dyes are highly toxic and mutagenic, and decrease light penetration and photosynthetic activity, causing oxygen deficiency and limiting downstream beneficial uses such as recreation, drinking water, and irrigation [4].

It has been shown that azo and nitro-compounds are reduced in sediments [5] and in the intestinal environment [6], resulting in the regeneration of the parent toxic amines [3]. Azo dyes and some of their N-substituted aromatic biodegradation products are toxic and/or carcinogenic and, hence, the dyes need to be treated as important environmental pollutants [7]. Bacterial azo dye biodegradation typically occurs in two steps. The first step involves a reductive cleavage of the dyes’ azo linkages, resulting in the generation of generally colorless but potentially toxic aromatic amines. The second one involves the degradation of the aromatic amines. Azo dye reduction usually happens under anaerobic conditions, while bacterial biodegradation of aromatic amines is mostly an aerobic process [8]. Bacteria can even degrade intermediate products of decolorization such as aromatic amines with the help of enzymes like hydroxylase and oxygenase [9], thus aiding in reducing lethal effects of azo dyes by the formation of non-toxic metabolites. These indicate the importance of a proper combination of anaerobic and aerobic bacterial communities in the azo dye treatment system. Moreover, it would be necessary to develop microbial consortia that harbor genes for the efficient degradation of mixtures of azo dyes and aromatic amines. The consortia will be quite useful for the bioaugmentation of the azo dye wastewater treatment system. The decolorization and COD removal of the azo dye wastewater in an anaerobic reactor packed with a pair of Fe-graphite plate electrodes were significantly higher than in a single anaerobic reactor and a single electrode reactor because of microbial richness and diversity stimulated by the electrochemical reaction on the electrodes [10]. Bioaugmentation of the microbial consortium SFC 500-1 showed a great potential for the treatment of tannery effluents under laboratory and field microcosm systems [11]. A mutualistic-symbiotic relationship between bioaugmented bacteria and soil indigenous microbes facilitated an enhanced detoxification of mixed dyes, leading to a sustainable approach for restoration of contaminated lands [12]. The bioaugmentation of hydrolysis acidification (HA) by a halophilic bacterial consortium facilitated an increase of bacterial diversity and dominant invasion of *Marinobacterium* which played a key role in azo dye decolorization [13]. An imperfect color removal with standard activated sludge system is related to changes in concentration and composition of dyes, and their toxicity, as well as sludge adaptation to such unstable conditions. Therefore, there is still a growing need in finding an effective and low-cost method for the removal of dyes from wastewater [4]. Industrial-scale studies for bioaugmentation have been applied to the target pollutants, including ammonia and polycyclic aromatic hydrocarbons (PAHs) [14].

Metagenomic analysis by using high-throughput sequencing methods may help to overcome the limitations of conventional methods of studying microbial community dynamics in the dye wastewater treatment process. The high-throughput sequencing analysis approach will provide a great opportunity and new insights to reveal the composition of microbial communities, diversity of functional genes, and enzymes responsible for the treatment of various wastewaters including tannery and textile wastewaters. There should be more studies for a better understanding of the degradation pathways where bioaugmentation is implemented. It will be also necessary to undertake bioaugmentation efficacy studies at full scale with test and control streams and evaluation of the economic viability of the technique [14]. Researchers have concentrated on designing numerous technologies [15] for the treatment of textile wastewater and also studied the changes in microbial community and diversity in the treatment systems. [16]. However, there have been no studies on high-throughput detection of microbial community profiles of environmental samples such as dye wastewater which have been severely affected by long-term exposure to improper discharge. Guo et al. (2013) and Krishnamoorthy et al. (2021) [17,18] used a metagenomic approach to unravel the community diversity and functional profiles within activated sludge from a full-scale SNPR WWTP and found that various key enzymes involved in metabolisms degrading various chemicals could be annotated in different treatments. However, it was hard to distinguish the effects of each divergent variable on microbial community in a full-scale dye wastewater treatment plant since a lot of uncontrollable or even undetectable influential factors were involved in such a pollutant-removing process [19]. Thus, it is necessary to evaluate adverse effects on the enzyme functions, variation on the microbial community structures and activities of the activated sludge under controllable processing conditions.

In this study, a full-scale dye wastewater treatment process was monitored and evaluated in terms of physicochemical parameters, sludge reduction, functional genes encoding enzymes responsible for degradation of various chemicals, as well as azo dyes and microbial community structures before and after bioaugmentation of the composite microbial culture CES-1 over more than 500 days. The intrinsic relationships among metabolic functional genes, the microbial community structures, and samples of different treatment stages and times were comprehensibly analyzed based on the metagenomic analysis technology. The obtained data appeared to successfully reveal a mechanistic basis of beneficial effects of CES-1 bioaugmentation on optimal treatment of the recalcitrant dye wastewater leading to sludge reduction (at least 22%) as well as meeting all requirements for the wastewater treatment standards at a full-scale system with good economic merit.

## 2. Materials and Methods

### 2.1. Description of Textile Dye Wastewater Treatment System and Its Operations

**Figure 1:**
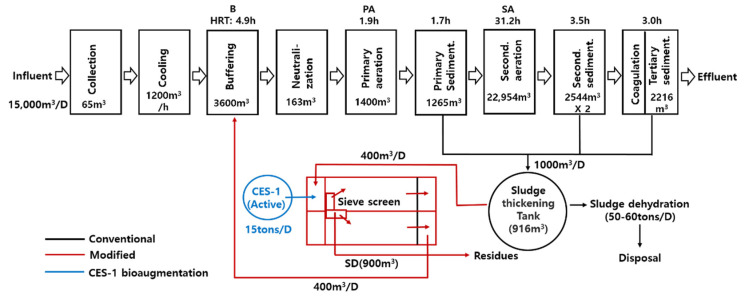
The full-scale treatment system of textile dye wastewater and its operational conditions. Sampling and monitoring sites: I, influent; B, buffering tank; PA, primary aeration tank; SA, secondary aeration tank; SD, sludge digestion tank. HRT hydraulic retention time. D, days.

The treatment system was a two-stage activated sludge process modified with the addition of a sludge digestion tank which was located at a dye wastewater treatment plant in Daegu City, South Korea. As shown in Figure 1, the process was essentially composed of multiple steps including the influent tank (I), the buffering tank (B), primary aeration tank (PA), primary sedimentation tank (PS), secondary aeration tank (SA), secondary sedimentation tank (SS), tertiary sedimentation tank (TS), and sludge digestion tank (SD). The wastewater treatment plant routinely treated 15,000 (m^3^ per day) of wastewater at 25–35 °C. One thousand (m^3^/day) of sludge was generated from primary, secondary, and tertiary sedimentation tanks and transported to the sludge thickening tank. Four hundred (m^3^/day) of the sludge was then transported into SD (900 m^3^) after filtration through a sieve screen (1 mm) and digested through bioaugmentation of the active culture of CES-1 (15 tons per day), and the remaining sludge (600 m^3^/day) was subjected to dehydration, generating dehydrated sludge (50–60 tons/D; 60% moisture). The digestion was performed at 35–42 °C and under 0.5–3.5 mg/L of DO for 54 h, and then the digested sludge was recycled into B. The composite culture CES-1 was initially enriched from the natural soils and was originally deposited by Bayo, Inc. (Jinju, Republic of Korea) as a proprietary culture (KCTC 12579BP) at the Biological Resource Center, Korea Research Institute of Bioscience and Biotechnology (Daejeon, Republic of Korea). The medium for CES-1 (powder form) includes the following ingredients: rice bran (35%, *w/w*), rice husk (35), oak sawdust (27), and minerals including illite, perlite, and vermicu-lite (3). The powder form culture was prepared by growing CES-1 inoculum (1%, *w/w*) in the medium (moisture, 55%) at 40 °C for 5–7 days. The liquid culture of CES-1 was prepared by inoculating 7 kg of CES-1 (powder form) into 1.0 m3 of water and growing it at 40 °C for 3 days under an aeration (50 L/min). The culture reached 108–109 (cells/mL) and was then diluted 30 times before being augmented into the sludge digestion tank (SD) at 0.1% (*v/v*) of the total influent treated daily (15,000 m^3^ × 0.1% = 15,000 L). Major genera of the liquid culture based on the pyrosequencing analysis were *Clostridium* (58.0%), *Lactococcus* (13.5), *Gluconacetobacter* (11.0), *Acidomonas* (4.9), *Klebsiella* (2.9), *Enterobacter* (1.9), *Lactobacillus* (1.2), *Chitinophaga* (1.1), *Dyella* (1.0), *Acidocella* (0.5), *Citrobacter* (0.5), *Lacibacter* (0.4), *Burkholderia* (0.4), and others (2.7). The hydraulic retention time for the whole wastewater treatment system was 46.2 h. Traditionally the sludge generated from the primary, secondary, and tertiary sedimentation tanks was transported to the sludge thickening tank and then the thickened sludge was dehydrated to reach 60% of moisture content (50–60 tons produced per day).

### 2.2. Monitoring the Water Quality of Dye Wastewater Treatment System

The samples were taken from the treatment plant and labeled with the sample name, month, date, and the year of the sample taken. The water quality of the dye wastewater treatment processes was monitored in terms of COD, T-N, T-P, NH_4_^+^, NO_2_^−^, NO_3_^−^, and suspended solid (SS). These parameters were measured following the Standard Methods for the Examination of Water and Wastewater [20] and using the wastewater analysis kits and HS-3300 water analyzer (Humas Inc., Daejeon, South Korea), and spectrophotometer (Model Optizen Pop, Mecasys Inc., Daejeon, South Korea) as previously described (Kim et al., 2014). pH was measured using a Neomet pH meter (Istek, Inc., Seoul, South Korea) and dissolved oxygen (DO) using a DO meter (Model Pro2030; YSI Inc., Yellow Springs, OH, USA). The color intensity was measured using platinum-cobalt (Pt-Co) analysis [20].

### 2.3. DNA Isolation from Samples and Sequencing of Metagenomes

DNA was isolated from CES-1 and 19 samples from the different treatment steps (I, B, PA, SA, and SD) during the five different periods (3 March 2017–29 June 2017; 10 March 2018–13 July 2018; and 26 October 2018). The names of the samples were described as treatment step_month and date_year. They were taken 67 days before augmentation (B_0303_17, PA_0303_17 and SA_0303_17), 50 days after augmentation (I_0629_17, B_0629_17, PA_0629_17, SA_0629_17, SD_0629_17), 300 days after augmentation (I_0310_18, B_0310_18, PA_0310_18, SA_0310_18, SD_0310_18), 425 days after augmentation (I_0713_17), and 531 days after augmentation (I_1026_18, B_1026_18, PA_1026_18, SA_1026_18, SD_1026_18). 0.25 g of biomass was collected by filtering each sample. To avoid the fragmentation of strands due to instability in the pH, the samples were washed with 0.1 M of EDTA. DNA extraction procedures have been shown previously [21], and detailed processes for library preparation, sequencing, and bioinformatics analysis of the metagenomes have been previously described [22]. Briefly, DNA extraction was performed on 1–2 milliliters of each sample, which was accomplished using the PowerSoil DNA Isolation Kit (Mo Bio Laboratories, Inc., San Diego, CA, USA). The samples were processed using TruSeq DNA LT Sample Prep Kit (Illumina, San Diego, CA, USA) and Paired-end sequencing (2 × 150 bp) was performed on an Illumina MiSeq system (Macrogen Inc., Seoul, South Korea) following the manufacturer’s protocol.

### 2.4. Contig Assembly and Binning of Metagenomes

The raw reads were trimmed using Trimmomatic (version 0.36) with a default option to filter low quality reads. The total reads and the GC% were shown in Table S1. Quality trimmed reads were used for taxonomic classification using Kraken2 with a standard database, and the selected genes and the encoded enzymes were listed in Table S2. This database consisted of the bacterial, archaeal, and viral genome sequences from RefSeq, along with the human genome and UniVec. Kraken2 results may mislead the results because the bacterial genome sequence of species (genus) level is nearly identical. Therefore, we re-estimated the relative abundance of each taxonomic level, using Bracken for more accurate taxonomy composition analysis.

### 2.5. Visualization of Metabolic Pathways and Microbial Communities

The statistical analysis of physical and chemical parameters for the wastewater was accomplished through one-way ANOVA using SPSS (IBM SPSS Statistics 25) with the significance *p* < 0.05. The visualization of bubble plots and analysis Spearman’s rank correlation analysis was conducted with R software using ggplot2 and pheatmap [23] and corrplot package [24]. The Procrustes correlation was analyzed from the VEGAN package and plot in ggplot in R and the *p*-value was obtained by recalculating and normalizing the abundance value. The simulated correlations between the pathways were confirmed if *p* < 0.05 [25]. The network analysis was done using Gephi with the whole abundance as a node based on a *p*-value (<0.05) and the modularity value as a cluster and edges. The co-occurrence correlation between the pathway gene and the microbial community presence was statistically checked and calculated [26].

## 3. Results and Discussion

### 3.1. Wastewater Treatment Monitoring during Bioaugmentation

The efficiency of the dye wastewater treatment 67 days before the bioaugmentation of CES-1 was shown in Table 1, which was used as a control for the treatments after the bioaugmentation. The removal efficiencies (%) for COD, T-N, T-P, SS, and color intensity were 94.9, 48.0, 91.6, 63.9, and 66.3, respectively. By the way, the removal efficiencies (%) for COD, T-N, T-P, SS, and color intensity 50 days after the bioaugmentation were 97.8, 62.6, 95.8, 63.4, and 77.9, showing the increase of COD, T-N, T-P, and color intensity removal efficiencies (%) by 2.9, 14.5, 4.2, and 11.6, respectively. This indicates that the bioaugmentation of CES-1 may be effective in facilitating dye wastewater treatment. Moreover, the removal efficiencies (%) for COD, T-N, T-P, SS, and color intensity 300 days (531 days) after the bioaugmentation were 95.5 (97.3), 80.9 (75.9), 96.2 (70), 44.1 (81.3), and 90 (94), respectively. Overall, the removal efficiencies for COD, T-N, T-P, SS, and color intensity after the augmentation were higher than the control (before augmentation).

The COD removal may be one of the main parameters used to prove sludge reduction and to understand its mechanism. Taking in the 531 days of augmentation, the removal rate of COD was 97.3% while the removal rate of COD before augmentation was 94.9%. A good sludge reduction (22%) was also observed at the time (Table 2). There was an increase in the removal rate (2.5 to 20%) throughout the bioaugmentation of CES-1 compared with the previous studies [26,27,28]. This process appeared to increase the total nitrogen removal efficiencies (62.6–80.9%) of the system compared with the control (48.0%). Removal of T-N appeared to be more active in the 0310_18 (80.9%) than before the bioaugmentation, and this kind of trend was maintained until 531 days after the bioaugmentation removal efficiencies of T-N (75.9–80.9%). The overall removal efficiencies of COD, T-N, NH_4_^+^, and T-P increased after the augmentation compared with before augmentation (Table 1). T-N removal is an important indication of the denitrification whose process requires electron donors derived from organic materials of which concentration can be estimated by COD. The removal rates of COD were also higher in the bioaugmented effluent samples. Therefore, the denitrification linked to T-N removal seemed to contribute to the concomitant COD removal that triggered the reduction of sludge which was the source of organic materials in the treatment system as shown previously [20,29].

Compliance with effluent nitrogen standards affects a wide variety of dyes. Nitrification is well known for its process instability due to the requirement for the close linking of the bacterial species responsible for different parts of the removal process [30]. Bioaugmentation has been shown to offer the potential to stabilize nitrification and to deal with transient treatment problems.

As with nitrogen, bioaugmentation has been demonstrated to have some success in the treatment [31] and an improved long-term stability of treatment systems for treating aromatic compounds under such bioaugmentation conditions was observed.

### 3.2. Comparative Analysis of Potential Different Metabolic Pathways Involved in Chemical Degradation Processes in the Treatment System

Besides understanding of the wastewater treatment processes in terms of dynamics of the chemical parameters, it will be highly worthwhile to try to understand the treatment process at a molecular biological level based on metagenome analysis of genes (enzymes) and microbial communities responsible for degradation of chemicals and its resulting sludge reduction. Four groups of the metabolic pathways were compared with each other, and the data showed that they appeared to be highly correlated with each other, indicating they are metabolically interrelated [28]. Most of the samples taken after bioaugmentation (I_0713_18, I_1026_18, B_1026_18, PA_1026_18, SA_1026_18, SD_1026_18) were highly clustered, while the sample before the bioaugmentation (B_0303_17). The augmentation of CES-1 appeared to allow some microbial communities to be selected strongly over others so that these metabolic pathways could be highly functional in the positive correlations.

**Figure 2:**
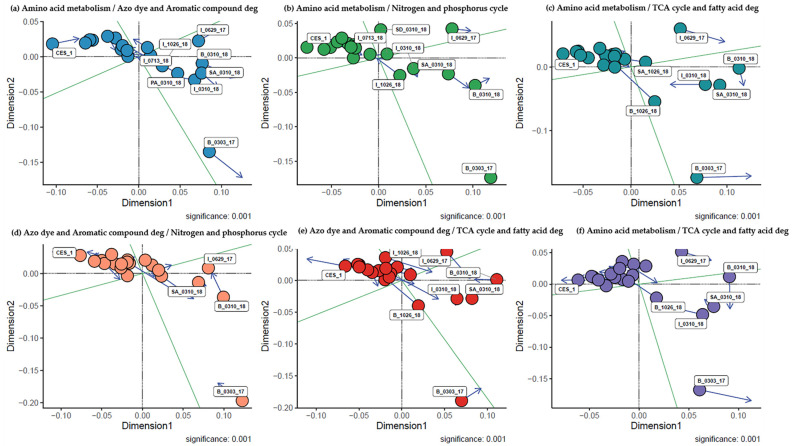
Procrustes analysis of the major potential degradation pathways in textile wastewater treatment system before and after bioaugmentation of CES-1 over the monitoring period (531 days). Blue arrow indicates the significant positive correlation between the two pathways (*p* < 0.05).

Procrustes analysis demonstrated a significant association between the ordinations of two metabolic pathways. In the association of azo dye and aromatic compound degradation, amino acid metabolism and nitrogen and phosphorus cycle with TCA cycle, B_1026_18, and SA_1026_18 got shifted towards the TCA cycle. Most of the samples from 300 days of bioaugmentation and CES-1 were correlated with azo dye and aromatic compound degradation, amino acid metabolism, and nitrogen and phosphorus cycle. The rest of the clustered samples were stretched towards the azo dye and aromatic compound degradation and amino acid metabolism. The results indicated that the samples 300 and 531 days after the bioaugmentation showed high interrelationships and continuities among pathways and stretch towards the TCA cycle (Figure 2a–f). Comparison of the Procrustes plot with chemical analysis revealed that the positive correlations of amino acid metabolism with nitrogen cycle and TCA cycle support the higher COD removal rate in 50 days, and 531 days of augmented samples. It was assumed that the degradation of amino acid and nitrogen cycle might facilitate the enhancement of COD removal [21,32]. The presence of the TCA cycle in B_1026_18, SA_1026_18, and B_0629_17 indicates the carbon source utilization by microbial communities, which completes the cycle of sludge reduction [29].

### 3.3. Dynamic Changes of Functional Genes for Major Metabolic Pathways in Textile Dye Wastewater Treatment Process Undergoing Bioaugmentation of CES-1 over Time

To understand mechanisms of the degradation of azo dye and other accompanying chemicals, and sludge reduction at a molecular biological level, the metagenomic analysis was done by sequencing the whole microbial community DNA extracted from the twenty different treatment stages. Major functional genes were selected according to their relevant pathways, and their dynamic changes were analyzed using their abundances in all the samples over time.

**Figure 3:**
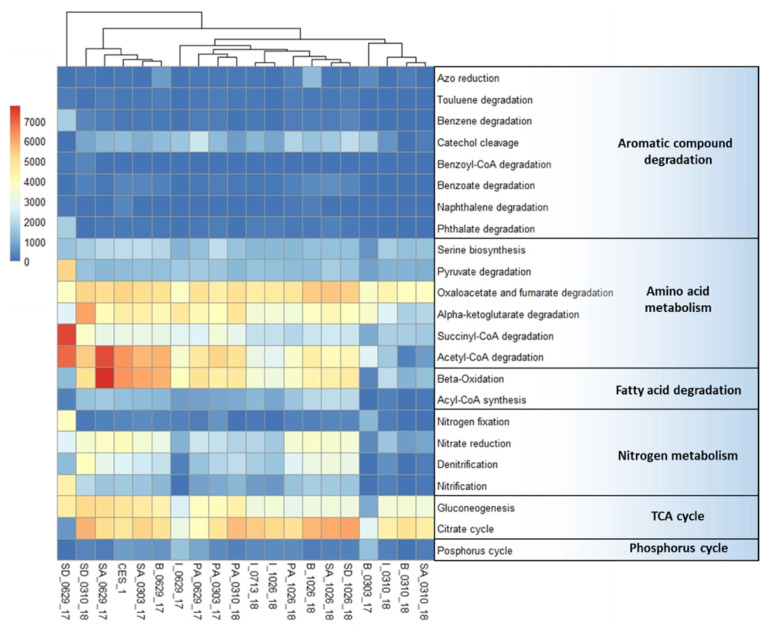
Heatmap analysis of the major potential degradation pathways and their modules in textile wastewater treatment system before and after bioaugmentation of CES-1 over the monitoring period (531 days).

The gene abundance profiles of major metabolic pathways in the treated samples were shown in Figure 3. Starting with aromatic compound degradation, the presence of genes involved in catechol cleavage was higher in most of the samples including CES-1. The catechol cleavage pathway was comparatively higher than the other pathways involved in aromatic compound degradation. According to Aghapour et al. [28], the catechol cleavage leads to the formation of succinyl-CoA, acetyl-CoA, and pyruvate. This requires the presence of genes involved in their pathways for amino acid metabolism. Abundances of the dominant amino acid degradation genes were higher in CES-1 and the samples that went through an early augmentation such as B_0629_17, SA_0629_17, and SD_0629_17. Abundances of the TCA cycle and fatty acid degradation genes were also higher in most of the samples. This may indicate the necessity of these pathways to provide the carbon sources for the growth of the microbial communities. In nitrogen metabolism, the nitrate reduction and the denitrification follow the same trends in 50 days and 531 days of bioaugmented samples. Supporting the higher presence of denitrification genes after the bioaugmentation, the nitrate reduction leads to a high rate of denitrification [22]. Analyzing the data for chemical parameters, the samples (531 days after augmentation) with a higher frequency of genes for degradation of amino acid, denitrification, and TCA cycle could have contributed to the sludge reduction (Table 2).

**Figure 4:**
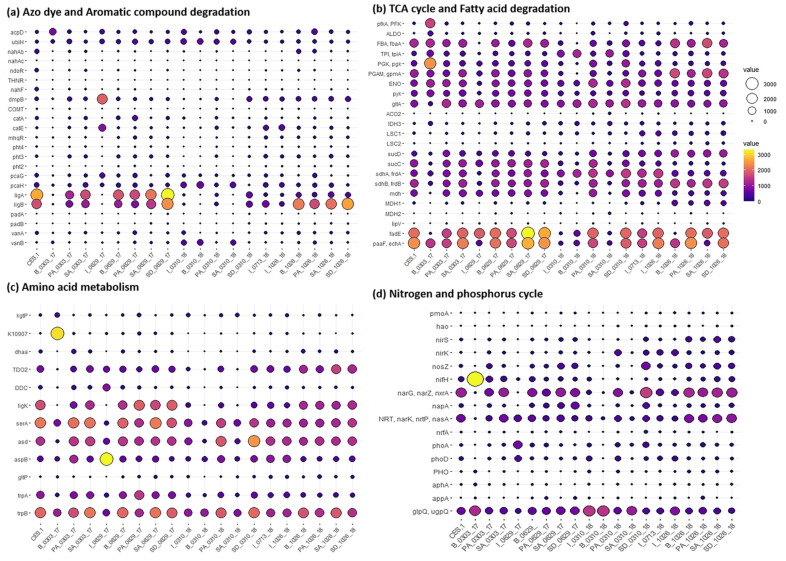
Abundance dynamics of genes encoding for enzymes involved in the major potential degradation pathways in textile wastewater treatment system before and after bioaugmentation of CES-1 over the monitoring period (531 days).

Unexpectedly, azo dye reductase gene (*acpD*) showed a lower frequency in most of the samples although its removal efficiencies increased as the augmentation progressed (from 78 to 94%) (Figure 4a; Table 1). Aromatic compound degradative genes *ligA* and *ligB* (encoding protocatechuate 4,5-dioxygenase alpha chain and beta chain, respectively) were dominantly carried in the augmented culture CES-1 and all the 2017 samples except B_0303_17 and I_06_29_17, which were taken before bioaugmentation and in the early stage of bioaugmentation. These genes almost disappeared in all the samples taken 300 days after the augmentation (10 March 2018). However, *ligB* gene reappeared in all samples 531 days after the augmentation (26 October 2018) except the influent, indicating the stabilized maintenance of this gene in the steady-state of the treatment system. The cleavage of azo bonds (-N=N-) and the replacement of hydroxyl group of *ortho*-carboxyl group with F- (fluorobenzene) lead to the breaking of benzene ring and the compound degraded into toxic-less metabolites such as, 2-methyl-propionic acid [33,34]. The *ligA* and *ligB* genes might support the asymmetric cleavage of the C–N bond, removing N in the aromatic ring, and yielding several intermediate products, such as 9-octadecenamide, (Z)- and phenylethyl alcohol, and *p*-xylene, and the breakage of benzene ring opening and the transformation of long-chain compounds into low-molecular-weight and less-toxic stable products, such as butanoic acid, 3-methyl-, and 4-methyl-2-oxovaleric acid. A recent study has demonstrated the successful decomposition of azo dyes into aromatic amines due to sufficient electron donor source [32]. Most of the functional genes involved in TCA cycle (*fbaA*, *tpiA*, *pgk*, *gpmA*, *ENO*, *pyk*, *gltA*, *sucD*, *sdhA*, *sdhB*, and *mdh*) and fatty acid degradation pathways were observed in most of the samples before and after the augmentation (Figure 4b). Moreover, the fatty acid degradative genes encoding acyl-CoA dehydrogenase and enoyl-CoA hydratase were essentially more dominant over the whole experimental period. This indicates that most of the genes for TCA cycle and fatty acid degradation appeared to be widely distributed in the treatment system regardless of bioaugmentation of CES-1. Azo dye degradation requires electrons generated from the fatty acid degrading genes and TCA cycle [35]. In terms of amino acid metabolism, the dominant genes were *ligK*, *serA*, *asd*, and *trpB*, which were found in most of the samples over the observation period (Figure 4c). The amino acid metabolic genes could be non-specifically functioning regardless of the bioaugmentation. As to nitrogen and phosphorus metabolism, the genes responsible for denitrification including *narG*, *narZ*, and *nxrA* were relatively dominant in most samples except influent samples and some from buffering (B_0303_17 and B_0310_18) (Figure 4d). In previous studies, metagenome for a full-scale bioreactor in Aalborg, Denmark (DK), that performed nitrogen removal in addition to phosphate removal was reported [36]. These observations are corresponding with the lower concentration of nitrate and nitrate in 0310_18 and 1026_18. However, the nitrite reductase gene (*nrfA*) involved in the treatment showed 2–4 times lower abundance than the nitrate reductase and denitrification genes (*nirS*, *nosZ*, *narG*, *narZ*, and *nxrA*) in the later bioaugmentation, indicating that the treatment system was operated under the conditions favoring denitrification. Total nitrogen (T-N) and COD simultaneously decreased as the augmentation continued over the period, whose phenomena were typically observed in denitrification process. This indicates the COD was the main electron donor source for the removal of total nitrogen [37]. Therefore, these denitrification genes appeared to be functioning in the system under an influence of the CES-1 augmentation. The denitrification also seemed to contribute to the sludge reduction of the system by 22% about 17 months after the augmentation.

### 3.4. Dynamic Changes of Microbial Community Structures in Textile Dye Wastewater Treatment Process under Bioaugmentation of CES-1 at Different Treatment Stages over Time

The families such as Aeromonadaceae were dominant 67 days before and 50 days after bioaugmentation while Bulkholderiaceae, Commamonadaceae, Dermatophilaceae, and Bulkholderiales_noname were dominant 300 and 531 days after the augmentation shown in Figure S1. Since Aeromonadaceae were highly dominant in samples before bioaugmentation as well as in CES-1, the family also became dominant in samples 50 days after bioaugmentation. Burkholderiaceae was dominant in 300 and 531 days after the augmentation. In addition, *Sphingobium*, *Sulfitobacter*, *Mesorhizobium*, *Acetobacter*, *Thiomonas*, *Alicycliphilus*, and *Burkholderia* were generally dominant before and in the early stage of augmentation (Figure S2), but they all became much less dominant in the later stages of augmentation. Several genera including *Burkholderia* and *Sphingobium* utilized the azo and aromatic compounds like benzene, pyrene, and flourene as a sole carbon and energy source [33]. *Acetobacter* and *Glucanocetobacter* were dominant in BM-S-1 that seemed to play an important role in amino acid degradation pathways [30]. Our data were in agreement with the findings of previous studies in the dominance of Proteobacteria in dye wastewater samples [34,38]. Proteobacteria are generally known to have an important role in the degradation of organic pollutants such as nitrogenous, phosphorus and aromatic compounds. Denitrification can be mediated by bacteria, archaea, and eukaryotes, but most of the denitrifiers belong to the phylum [39].

**Figure 5:**
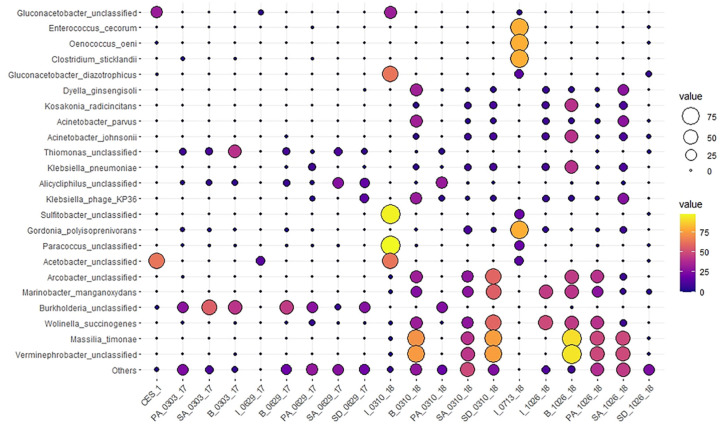
Abundance dynamics of microbial communities (species level) in textile wastewater treatment system before and after bioaugmentation of CES-1 over the monitoring period (531 days).

At the species level, *Thiomonas_unclassified*, *Alicycliphilus_unclassified*, and *Burkholderia_unclassified* were mostly dominant 67 days before augmentation and 50 days after augmentation, but they were significantly diminished in the later stages of augmentation (Figure 5). Although these species seemed to be transient, they may play certain roles in degradation of pollutants in a “low key” mode. It has been reported that *Marinobacter sp.* have an ability to degrade the O-H and N-H stretching corresponding to aromatic hydroxyl and amino groups in azo dye color index Direct Blue-1 [40]. The microbial community data indicate that augmentation of CES-1 carrying *Gluconacetobacter_unclassified* and *Acetobacter_unclassified* as dominant species allowed a good selection of a greater number of species, leading to a higher diversity index (Shannon indices: 2.11–3.25) in the later stages of augmentation compared with before augmentation and at the early stage of augmentation (Shannon indices: 1.24–2.61). A large number of unclassified sequences were found in dye wastewater [41], suggesting that a wide variety of novel species may inhabit in the dye wastewater treatment system [42]. It is suggested that many degrading microbes appeared to be enriched in B, PA, SA, and SD in the later stages of augmentation because of long term bioaugmentation of CES-1 into SD. *A. johnsonii* and *A.* *parvus* carried genes for azoreductase and proved to be efficient in degrading textile effluents [43,44]. Furthermore, *Pseudomonas* species carries genes for at least three different azoenzymes *azoR1*, *azoR2*, and *azoR3* [45]. *Sphingomonas* has genes for several azoreductases and has performed both successful dye degradation and mineralization of metabolites [46]. There were many less microbial communities present before bioaugmentation due to the presence of high concentrated azo compounds in the wastewater [18]. This might have resulted in the decrease of microbial populations and subsequently reducing enzyme activities [47]. In this study, however, there was a drastic increase in the abundance of microbial communities (by 75%) in B, SA, and SD after 300 days of bioaugmentation.

**Figure 6:**
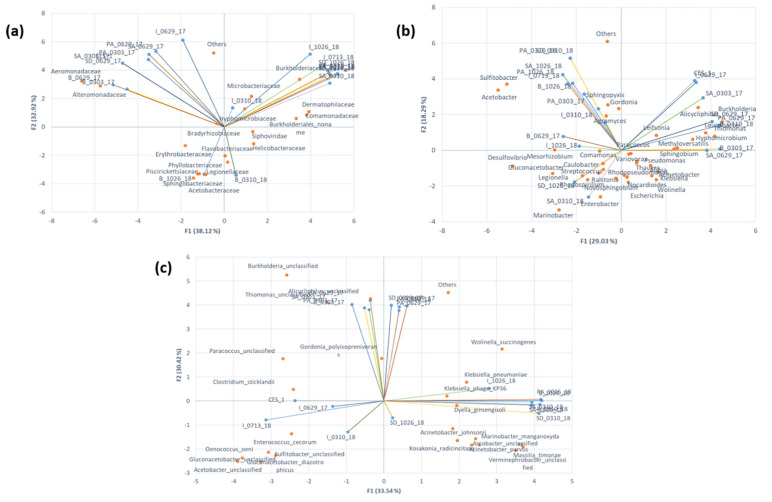
Principal component analysis (PCA) of microbial community with linear combinations of the variables sorted to different quantitative variables. The arrow lines represent the correlation coefficient between the principal component scores and parameter. Treatment stages indicated in the blue dot, denitrification genes and degradation enzymes indicated in orange dot. PCA of the relative abundance at family level (**a**); PCA of the relative abundance at genus level (**b**); PCA of the relative abundance at species level (**c**).

On the other hand, a PCA indicated a transition in the composition of the microbiota at the species level in the different treatment systems. The composition of the bacterial community in the family level including Acetobacteraceae, Sphingobacteraceae, and Flavobacteriaceae was affiliated with B_1026_18 and B_0310_18 (Figure 6a). At the species level, B_1026_18 and B_0310_18 were diverged from negative correlation and joined with the rest of the samples from 1026_18 and 0310_18 in the positive correlation including *Acinetobacter_johnsonii*, *Wolinella_succingenes*, and *Klebsiella_pneumoniae*. The species from Acetobacteraceae, Sphingobacteraceae, and Flavobacteriaceae have stayed in negative correlation (Figure 6c). Considering the range distribution values in both F1 and F2 dimension, the family and species level had significance. There is no universal rule for the estimation of the number of PCs [48]. It was assumed that there was a common correlation between the CES-1 and the 50 days of bioaugmented samples indicating a strong influence of CES-1 in 0629_17 samples. The positive correlation of 1026_18 and 0310_18 showed its significance among the treatments. Additionally, Figure 6c showed a higher presence of *Acinetobacter* *parvus* and *Acinetobacter* *johnsonii* with 1026_18 samples. Previous studies mentioned the extraction of azoreductase, NADH-DCIP reductase, laccase, and lignin peroxidase in *Acinetobacter sp.* [49]. The higher abundance of *Acinetobacter sp.* in the B_0310_18 and SA_1026_18 shows its contribution to degrade azo dye through several enzymatic mechanisms for the nonspecific reductive cleavage of azo linkage [36]. An increase of potential species present in the bioaugmented samples indicates its enzyme activity associated with their contribution to dye degradation.

### 3.5. Analysis of Relationships between the Microbial Community Structures and Major Degradation Pathways Involved in the Textile Dye Treatment Process under Bioaugmentation

**Figure 7:**
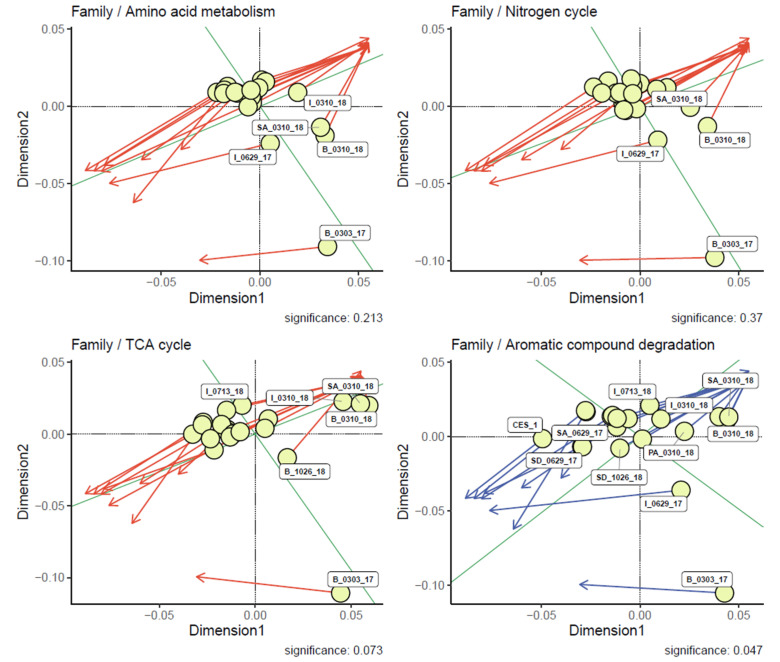
Procrustes analysis of the major potential degradation pathways and different microbial families in textile wastewater treatment system before and after bioaugmentation of CES-1 over the monitoring period (531 days). Blue arrow indicates the significant positive correlation between the microbial family and aromatic compound degradation (*p* < 0.05).

The Procrustes plot showed a positive correlation only between the microbial family communities and the azo dye/aromatic compound degradation pathway (Figure 7). The correlation between the pathway with genus and species does not show the significance (Figures S3 and S4) As shown in Figure 2, B_0310_18 and SA_0310_18 have higher abundance and positive correlations between azo dye/aromatic compound degradation and all the other degradation pathways (amino acid metabolism, TCA cycle and lipid degradation, and nitrogen/phosphorus cycles). The relative abundance of Burkholderiaceae increased in the later stages of bioaugmentation may prove that the microbial communities from the family can utilize the aromatic compound as a sole carbon and energy source [33,34].

### 3.6. Time-Course Network Analysis among Microbial Communities, Metabolic Pathways Involved in the Degradation of Azo Dye and Other Compounds

**Figure 8:**
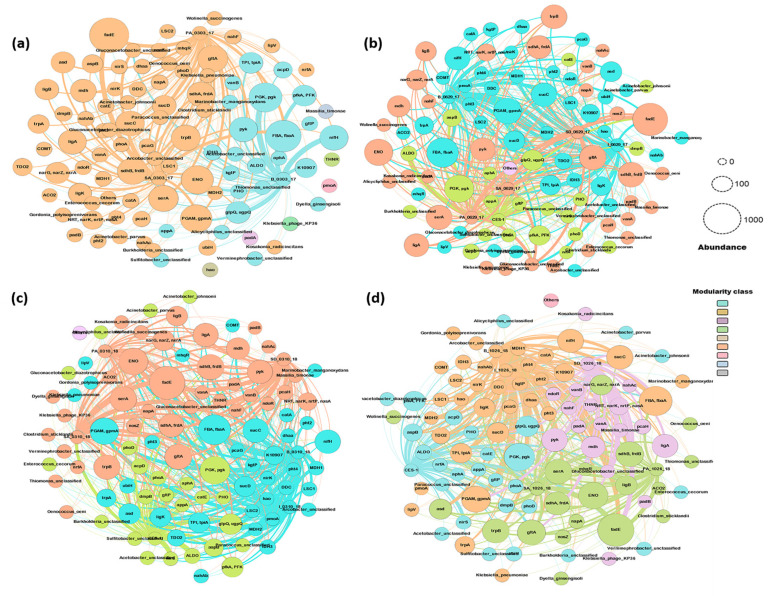
The comprehensive network analyses of the microbial species and their genes involved in the major potential degradation pathways, depending on the samples of the four different time courses in textile wastewater treatment system. (**a**) 67 days before bioaugmentation of CES-1; (**b**–**d**) 50, 300, and 531 days after bioaugmentation, respectively.

Based on previous studies [21,29,34], it was hypothesized that the bioaugmentation of potential microorganisms in dye wastewater reduced the sludge and increased the frequency of genes degradative for toxic organic and inorganic compounds. In general, primary aeration and secondary aeration of all the time frames were formed in a common cluster with *fadE*, *gltA*, *ENO*, *ligA*, *ligB*, and most of genes for the nitrogen and phosphorus cycle metabolic pathways. *Gluconacetobacter_unclassified* and *PGAM*, *gpmA* gene had a strong presence in all the samples of before bioaugmentation (Figure 8a). The formation of clusters from the sample before bioaugmentation indicates that the specific genes (*FBA*, *PGK*, *pyk*, *nifH*, and *glpQ*) and species (*Gluconacetobacter_unclassified*) used to play a major role throughout the treatment process. This weak connection among the treatment stages proves the fluctuations of microbial presence and the gene occurrence in the treatment time period.

The sample network 50 days after bioaugmentation had 99 nodes and 434 edges with the modularity value of 0.29. *ligA* and *trpB* had a strong connection with I_0629_17 and formed a cluster with PA_0629_17, SA_0629_17, SD_0629_17. Most of the dominant genes including K10907, *ligK*, *dhaa* associated with *Acinetobacter_parvus*, *Acetobacter_unclassified*, *Gluconacetobacter_diazotrophicus*, and *Marinobacter_manganoxydans* was clustered with PA_0629_17, SA_0629_17, SD_0629_17. The lignin peroxidase and azo reductase enzyme present in *Acinetobacter sp.* involves in a dye degradation [49]. The *pht* (phthalate 4,5-dioxygenase) and K10907 (aminotransferase) genes connected with *Acinetobacter* sp. and *Marinobacter* sp. in the cluster with B_0629_17 showed the connection between the azo and aromatic compound degradation and amino acid metabolic pathway in a specific microbial community in the buffering treatment [13].

In the samples 300 days after the augmentation, CES-1 got closely connected (strong edge between the samples) with SA_0310_18. This proved the impact of CES-1 on other samples, including SA. Both CES-1 and SA were a dominant host for azo dye and aromatic compound degradation genes including *ligA*, *ligB*, *catA*, *PHO*, *acpD*, etc. I_0310_18 and B_0310_18 were grouped and had a major connection with PA_0310_18 and SA_0310_18, where it clustered with SD_0310_18 with the total of 102 nodes and 431 edges with the modularity value of 0.32. Most of the communities came under these two groups. Comparing with the samples 50 days after bioaugmentation, there was little change in the clusters. *Gluconacetobacter_unclassified* was a common host for most of the genes and highly shared its edges with all the samples (Figure 8c). Most of the genes stayed in the same cluster until 300 days of bioaugmentation. CES-1 forms a separate cluster in all the time frames but shares most of its genes and communities with B, PA, SA, and SD. SD had a strong connection in the 50 days of bioaugmented samples, and the gene clusters from SD became more abundant in B and SA in later stages. *Actinobacter_parvus* remained in the CES-1 cluster but changed its edge from B to PA, SA, and SD. *ALDO*, *nrfA*, *appA* were the unchangeable clusters of CES-1. *Paracoccus_unclassified*, *Suffitobacter_unclassified*, *Burkholderia_unclassified* commonly carried the high abundant genes like *pgk*, *pfkA*, *ALDO*, *glpQ* from TCA cycle, *nirS*, *nrfA*, and *PHO* from the nitrogen cycle. *Actinobacter_johnsonii* became a host for *catE* from the aromatic compound degradation pathway in CES-1.

The final network formed with 102 nodes, 463 edges, and modularity value of 0.361. *fadE*, *ENO*, and *Glucanobacter_unclassified* were clustered with PA_1026_18 and commonly present in all the bioaugmented samples after 531 days (Figure 8d). After 531 days of bioaugmentation, the SD_1026_18 was separated from PA_1026_18 and SA_1026_18 and formed a tight connection with nitrogen, phosphorus, and TCA cycle genes in association with *Massilia_timonae, Gluconacetobacter_unclassified*, and *Kosakonia_radicincitans*. The overall separation and higher connection with CES-1 proved the dominance of CES-1 in the samples. The microbial community pattern may also depend on the fact that most bacteria with azoreductase (*acpD*) and catechol cleavage (*catA* and *catB*) use electron shuttles to degrade the azo bond and oxygen inhibits this process by being a more potent electron acceptor [50]. Moreover, since the concentration of COD and T-N decreased substantially with the higher abundance and correlation of *Wolinella_succinogenes*, *Klebsiella_pneumoniae*, and *Acinetobacter_johnsonii* in the 300 and 531 days of bioaugmented samples, the microbial communities could have utilized COD (derived from organic materials) and T-N as carbon and nitrogen sources [51]. As previously mentioned, *Acinetobacter* may play a major role in dye degradation [41]. The network analysis can prove the potential occurrence of degrading metabolic genes in the specific species present in CES-1 and the CES-1 bioaugmented samples. The azo dye degradative gene (*acpD*) from CES-1 was present in all the treatment timelines. The gene was highly dominant in the buffering sample (B_1026_18) carrying *Glucanacetobacter_unclassified* after 531 days of bioaugmentation. Compared with the three samples before bioaugmentation, buffering samples (B_0629_17, B_0310_18, and B_1026_18) established a major cluster and were highly related to most of the pathway genes and the microbial communities. Buffering carries the biomass of carbon and nitrogen sources, and other inorganic nutrients recycled from the sludge digestion tank (Figure 1). This implies that the degradative pathway genes from the microbial communities highly occurred in B after bioaugmentation. CES-1 was continuously augmented into SD so that the microbial communities and the genes from the culture could play a role in digestion of the sludge of SD. The digested sludge was then recycled into B together with the CES-1 communities and their genes which were subsequently flowed into PA and SA, indicating a strong influence of CES-1 on the whole treatment system. The sludge reduction caused by bioaugmentation was observed over the experimental period (Table 2). The metagenomic network analysis helps us to understand molecular-based functions of degradative pathway genes in specific microbes in the treatment process. Recently, network analysis has been widely used to explore potential microbes and their degradative pathway genes different environments [25]. The higher consistent results mentioned above indicated that the network analysis might be a reasonable and powerful tool to provide new insights and useful biomarker in the complex environmental examples.

## 4. Conclusions

In this study, effects of bioaugmentation of the composite microbial culture CES-1 on a full scale dye wastewater treatment process were investigated in terms of water quality, sludge reduction, dynamics of microbial community structures, and their functional genes responsible for degradation of various chemicals. The removal efficiencies for COD, T-N, T-P, SS, and color intensity after the augmentation were higher than before augmentation. The denitrification linked to T-N removal seemed to contribute to the concomitant COD removal that triggered reduction of sludge. Azo dye and aromatic compound degradation and other downstream pathways were highly metabolically interrelated. Augmentation of CES-1 allowed an increase of higher diversity index in the later stages of augmentation. *Acinetobacter* sp., *Glucanacetobacter* sp., *Arcobacter* sp., and *Marinobacter manganoxydans* might have utilized COD and T-N as carbon and nitrogen sources, leading to denitrification. The network analysis turned out to be a reasonable and powerful tool to provide new insights and useful biomarkers for the complex environmental conditions, such as dye wastewater treatment system undergoing a bioaugmentation. Moreover, functional analyses of the microbial communities using metatranscriptomics and real time PCR techniques will be necessary to better understand the mechanisms of this bioaugmentation technology. This novel technology may also contribute to full-scale treatments of sewage wastewater, pulp wastewater, livestock wastewater, food wastewater, and industrial malodors.

## Figures and Tables

**Table 1:** Characteristics of the dye wastewater of the different treatment stages before and after the bioaugmentation of the composite microbial consortium CES-1.

	3 March 2017 (67 Days Before Augmentation)			29 June 2017 (50 Days After Augmentation)					10 March 2018 (300 Days After Augmentation)					26 October 2018 (531 Days After Augmentation)				
	Influent [I]	Effluent [E]	Removal Rate (%)	Influent [I]	Buffering Tank [B]	Effluent [E]	Sludge Digestion [SD]	Removal Rate (%)	Influent [I]	Buffering Tank [B]	Effluent [E]	Sludge Digestion [SD]	Removal Rate (%)	Influent [I]	Buffering Tank [B]	Effluent [E]	Sludge Digestion [SD]	Removal Rate (%)
COD (mg/L)	584 ± 8.5 ^c^	20.6 ± 0.4 ^a^	94.9	674 ± 51.6 ^d^	466 ± 24.7 ^c^	14.5 ± 0.3 ^a^	141.5 ± 57 ^b^	97.8	472 ± 3.5 ^d^	440 ± 13 ^c^	21.1 ± 0.4 ^a^	2228 ± 3.5 ^e^	95.5	237 ± 79 ^b^	2112 ± 65 ^d^	7.3 ± 0.9 ^a^	1678 ± 76 ^c^	97.3
T-N (mg/L)	36.2 ± 1.3 ^b^	18.8 ± 0.8 ^a^	48	23 ± 0 ^c^	33.4 ± 0.8 ^d^	8.6 ± 0.4 ^b^	22.5 ± 0.1 ^c^	62.6	51.2 ± 0.1 ^c^	48.8 ± 2.7 ^c^	9.7 ± 1.4 ^a^	30.5 ± 1.3 ^b^	80.9	89.4 ± 0.74 ^e^	81.4 ± 0.96 ^d^	21.5 ± 3.1 ^a^	62.3 ± 0.5 ^c^	75.9
T-P (mg/L)	5.1 ± 0.5 ^b^	0.43 ± 0.01 ^a^	91.5	2.4 ± 0.1 ^b^	1.9 ± 0 ^b^	0.1 ± 0 ^c^	6.0 ± 1.2 ^a^	95.8	3.3 ± 0.1 ^c^	4.5 ± 0.4 ^d^	0.1 ± 0.04 ^a^	1.9 ± 0.09 ^b^	96.2	7 ± 0.1 ^d^	3.9 ± 1.2 ^d^	2.1 ± 0.41 ^bc^	2.9 ± 0.56 ^c^	70
NH_3_ (mg/L)	14.8 ± 0.5 ^b^	8.9 ± 0.4 ^a^	43.6	10.5 ± 0.1 ^c^	9.6 ± 0.1 ^b^	6.1 ± 0.2 ^a^	9.5 ± 0.1 ^b^	41.9	7.2 ± 0.9 ^c^	9.5 ± 0.5 ^c^	4.1 ± 0 ^a^	10.8 ± 0.5 ^c^	48.89	16 ± 0.2 ^e^	13.9 ± 0.1 ^c^	6 ± 0.7 ^a^	17.9 ± 0.4 ^f^	62.5
NO_2_^−^ (mg/L)	1.7 ^c^	0.3 ^a^	82.3	1.43 ^d^	1.01 ^b^	1.4 ^c,d^	0.9 ^b^	2	1.92 ^c^	0.95 ^b^	0.17 ^a^	0.9 ^b^	91.1	2.5 ± 0.6 ^c^	1.6 ± 0.23 ^b^	0.4 ± 0.1 ^a^	2.8 ± 0.1 ^c^	84
NO_3_^−^ (mg/L)	7.7 ± 0.2 ^c^	0.8 ± 0.07 ^a^	89.6	7.1 ± 0.5 ^d^	5.4 ± 0 ^c^	2.8 ± 0.4 ^b^	14.6 ± 0.2 ^e^	60.5	3.5 ± 0.14 ^b^	4.6 ± 0.6 ^c^	1.8 ± 0.4 ^a^	1.5 ± 0.03 ^d^	47.7	11.7 ± 0.4 ^c^	7.74 ± 0.2 ^b^	2.1 ± 0.2 ^a^	3.59 ± 0.5 ^a^	82
PO_4_ ^3−^(mg/L)	3.4 ± 0 ^c^	0.2 ± 0.01 ^a^	94.1	3.54 ± 0.2 ^d^	4.3 ± 0.05 ^e^	0.16 ± 0 ^a^	3.41 ± 0.05 ^c^	95.4	3.9 ± 0.3 ^c^	0.8 ± 0.1 ^a^	0.2 ± 0.01 ^a^	4.3 ± 0.6 ^c^	94.3	5 ± 0.3 ^e^	1.5 ± 2.8 ^b^	0.9 ± 0.04 ^a^	0.0 ± 0.2 ^d^	82
SS (mg/L)	-	-	-	123	-	45	-	63.4	776	-	97	-	87.5	478	**-**	**-**	89	81.3
Pt-Co(PCU)	-	-	-	347.9 ± 0.2	-	-	76.7 ± 1.1	77.9	305.1 ± 1.3	-	-	30.4 ± 1.5	90	361.1 ± 0.4	**-**	**-**	21.4 ± 0	94

Based on buffering replacing influent and secondary sedimentation replacing effluent. “^a,b,c,d,e,f^” alphabetic markings in ascending order are based on the Duncan test.

**Table 2:** Effect of CES-1 bioaugmentation on the sludge reduction in the full-scale dye wastewater treatment plant.

Sampling Period	Influent (M^3^)	Influent	Effluent	Sludge	Total Influent	Sludge	Sludge Reduction ***
				(ton)		Per ton of Influent	%
		COD	COD		COD	COD	
		(g/m^3^)	(g/m^3^)		(ton)		
2017. 3 *	411,588	664	34	1690	273	6.18	−
2018. 10 **	385,158	560	33	1007	217	5.00	22

* Monthly average before bioaugmentation; ** Monthly average after bioaugmentation; *** Sludge reduction per ton of influent COD.

## Data Availability

Not applicable.

## Supplementary Materials

The following are available online at https://www.mdpi.com/article/10.3390/microorganisms9071503/s1, Figure S1. Changes in the abundance of the microbial communities over time at the family level after bioaugmentation of the complex microbial consortium CES-1. Figure S2. Changes in the abundance of the microbial communities over time at the genus level after bioaugmentation of the complex microbial consortium CES-1. Figure S3. The Procrustes plot analysis of microbial community (Genus level) with different degradation pathways. The positive dimension is a nonnegative number, representing the credibility of the cluster results. Blue arrow indicates the significant positive correlation parameters (*p* < 0.05). Figure S4. The Procrustes plot analysis of microbial community (species level) with different degradation pathways. The positive dimension is a nonnegative number, representing the credibility of the cluster results. Blue arrow indicates the significant positive correlation parameters (*p* < 0.05). Table S1. Collection coordinates and metagenomic samples from the textile dye wastewater plant. Table S2. Genes and enzymes responsible for the degradation of zo dyes and other chemicals in the textile dye wastewater.
