# Supplementary material for: Metagenomic Insight of a Full Scale Eco-Friendly Treatment System of Textile Dye Wastewater Using Bioaugmentation of the Composite Culture CES-1
Source: Microorganisms. 2021 Jul 14;9(7):1503. doi: 10.3390/microorganisms9071503 (PMC8306160; doi:10.3390/microorganisms9071503)
Supplement: Supplementary file 1 [file microorganisms-09-01503-s001.zip › microorganisms-1272634-supplementary.pdf]

Table. S1. Collection coordinates and metagenomic samples from the textile dye wastewater plant

Samples	Total reads (bp)	GC(%)	AT(%)	Q20(%)	Q30(%)
CES_1	27,241,698	54.28	36.44	97.68	92.18
B_0303_17	31,658,964	64.17	37.65	97.38	93.25
PA_0303_17	36,985,624	67.29	37.59	98.39	93.48
SA_0303_17	37,658,742	67.54	37.42	93.46	94.61
I_0629_17	39,028,159	63.24	35.91	94.65	95.48
B_0629_17	43,645,559	67.51	36.18	94.06	91.26
PA_0629_17	48,942,653	66.58	34.44	94.13	92.18
SA_0629_17	45,451,628	66.04	37.52	96.56	93.37
SD_0629_17	44,448,571	67.98	36.37	96.27	93.68
I_0310_18	48,741,590	62.19	37.81	98.69	95.67
B_0310_18	49,077,062	64.69	35.31	98.63	95.46
PA_0310_18	49,372,460	59.77	40.23	98.61	95.46
SA_0310_18	49,277,998	58.25	41.77	98.75	95.9
SD_0310_18	49,514,724	54.81	45.18	98.48	95.26
I_0713_18	36,995,260	61.84	38.16	96.65	91.33
I_1026_18	34,172,512	60.51	39.49	94.54	91.26
B_1026_18	38,293,276	63.25	36.75	94.51	91.06
PA_1026_18	35,078,006	62.36	37.64	96.46	90.92
SA_1026_18	34,662,952	62.63	37.37	96.65	91.35
SD_1026_18	35,649,508	62.81	37.19	96.31	90.68

Table S2. Genes and enzymes responsible for the degradation of azo dyes and other chemicals in the textile dye wastewater

Genes for pathways	Enzymes encoded by the genes
**Azo and aromatic compound degradation**	
acpD	acpD, azoR; FMN-dependent NADH-azoreductase [EC:1.7.1.17]
ubiH	ubiH; 2-octaprenyl-6-methoxyphenol hydroxylase
nahAb	nahAb, nagAb, ndoA, nbzAb, dntAb; naphthalene 1,2-dioxygenase ferredoxin component
nahAc	nahAc, ndoB, nbzAc, dntAc; naphthalene 1,2-dioxygenase subunit alpha
ndoR	nahAa, nagAa, ndoR, nbzAa, dntAa; naphthalene 1,2-dioxygenase ferredoxin reductase component
THNR	THNR; tetrahydroxynaphthalene reductase
nahF	nahF; salicylaldehyde dehydrogenase
dmpB	dmpB, xylE; catechol 2,3-dioxygenase
COMT	COMT; catechol O-methyltransferase
catA	catA; catechol 1,2-dioxygenase
catE	catE; catechol 2,3-dioxygenase
mhqR	mhqR; MarR family transcriptional regulator, 2-MHQ and catechol-resistance regulon repressor
pht4	pht4; phthalate 4,5-cis-dihydrodiol dehydrogenase
pht3	pht3; phthalate 4,5-dioxygenase
pht2	pht2; phthalate 4,5-dioxygenase reductase component
pcaG	pcaG; protocatechuate 3,4-dioxygenase, alpha subunit
pcaH	pcaH; protocatechuate 3,4-dioxygenase, beta subunit
ligA	ligA; protocatechuate 4,5-dioxygenase, alpha chain
ligB	ligB; protocatechuate 4,5-dioxygenase, beta chain
padA	padA; phenylacetyl-CoA:acceptor oxidoreductase accessory protein
padB	padB; phenylacetyl-CoA:acceptor oxidoreductase
vanA	vanA; vanillate monooxygenase
vanB	vanB; vanillate monooxygenase ferredoxin subunit
**TCA_cycle**	
pfkA, PFK	pfkA, PFK; 6-phosphofructokinase 1
ALDO	ALDO; fructose-bisphosphate aldolase, class I
FBA, fbaA	FBA, fbaA; fructose-bisphosphate aldolase, class II
TPI, tpiA	TPI, tpiA; triosephosphate isomerase (TIM)
PGK, pgk	PGK, pgk; phosphoglycerate kinase
PGAM, gpmA	PGAM, gpmA; 2,3-bisphosphoglycerate-dependent phosphoglycerate mutase
ENO	ENO, eno; enolase
pyk	PK, pyk; pyruvate kinase
gltA	CS, gltA; citrate synthase

ACO2	ACO2; homoaconitase
IDH3	IDH3; isocitrate dehydrogenase (NAD+)
LSC1	LSC1; succinyl-CoA synthetase alpha subunit
LSC2	LSC2; succinyl-CoA synthetase beta subunit
sucD	sucD; succinyl-CoA synthetase alpha subunit
sucC	sucC; succinyl-CoA synthetase beta subunit
sdhA, frdA	sdhA, frdA; succinate dehydrogenase / fumarate reductase, flavoprotein subunit
sdhB, frdB	sdhB, frdB; succinate dehydrogenase / fumarate reductase, iron-sulfur subunit
mdh	mdh; malate dehydrogenase
MDH1	MDH1; malate dehydrogenase
MDH2	MDH2; malate dehydrogenase
**Fatty acid degradation**	
lipV	lipase
fadE	acyl-CoA dehydrogenase
paaF, echA	enoyl-CoA hydratase
**Amino acid metabolism**	
kgtP	kgtP; MFS transporter, MHS family, alpha-ketoglutarate permease
K10907	K10907; aminotransferase
dhaa	dhaa; 3-hydroxy-D-aspartate aldolase
TDO2	TDO2, kynA; tryptophan 2,3-dioxygenase
DDC	DDC, TDC; aromatic-L-amino-acid/L-tryptophan decarboxylase
ligK	ligK, galC; 4-hydroxy-4-methyl-2-oxoglutarate aldolase
serA	serA, PHGDH; D-3-phosphoglycerate dehydrogenase
asd	asd; aspartate-semialdehyde dehydrogenase
aspB	aspB; aspartate aminotransferase
gltP	gltP, gltT; proton glutamate symport protein
trpA	trpA; tryptophan synthase alpha chain
trpB	trpB; tryptophan synthase beta chain
**Nitrogen cycle**	
pmoA	pmoA-amoA; methane/ammonia monooxygenase subunit A
hao	hao; hydroxylamine dehydrogenase
nirS	nirS; nitrite reductase / hydroxylamine reductase
nirK	nirK; nitrite reductase
nosZ	nosZ; nitrous-oxide reductase
nifH	nifH; nitrogenase iron protein NifH
narG, narZ, nxrA	narG, narZ, nxrA; nitrate reductase / nitrite oxidoreductase, alpha subunit
napA	napA; periplasmic nitrate reductase NapA
NRT, narK, nrtP, nasA	NRT, narK, nrtP, nasA; MFS transporter, NNP family, nitrate/nitrite transporter
nrfA	nrfA; nitrite reductase (cytochrome c-552)
**Phosphorus cycle**	

phoA	alkaline phosphatase
phoD	alkaline phosphatase D
PHO	acid phosphatase
aphA	kanamycin kinase
appA	4-phytase / acid phosphatase
glpQ, ugpQ	glpQ, ugpQ; glycerophosphoryl diester phosphodiesterase

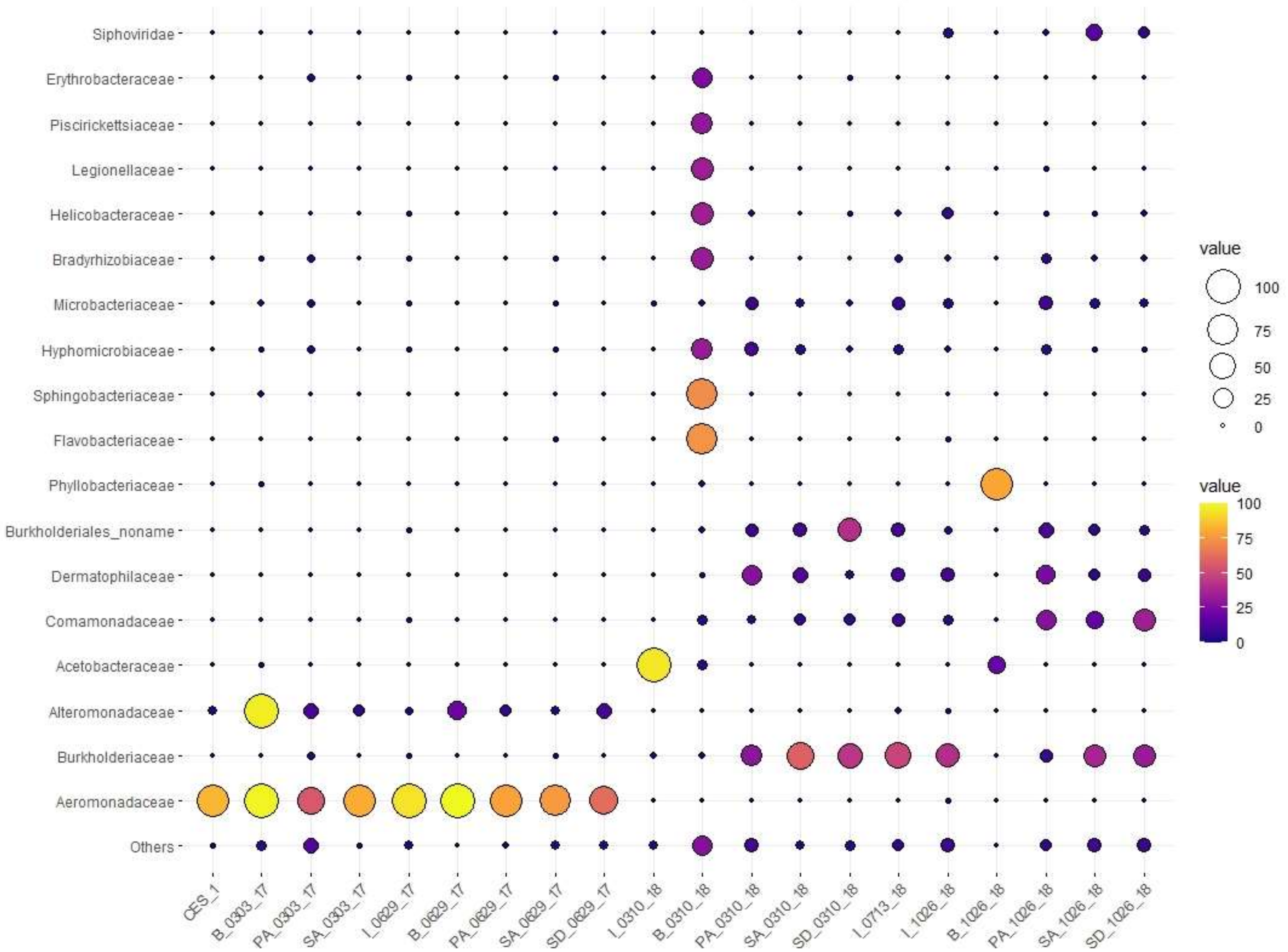

Figure S1. Changes in the abundance of the microbial communities over time at the family level after bioaugmentation of the complex microbial consortium CES-1

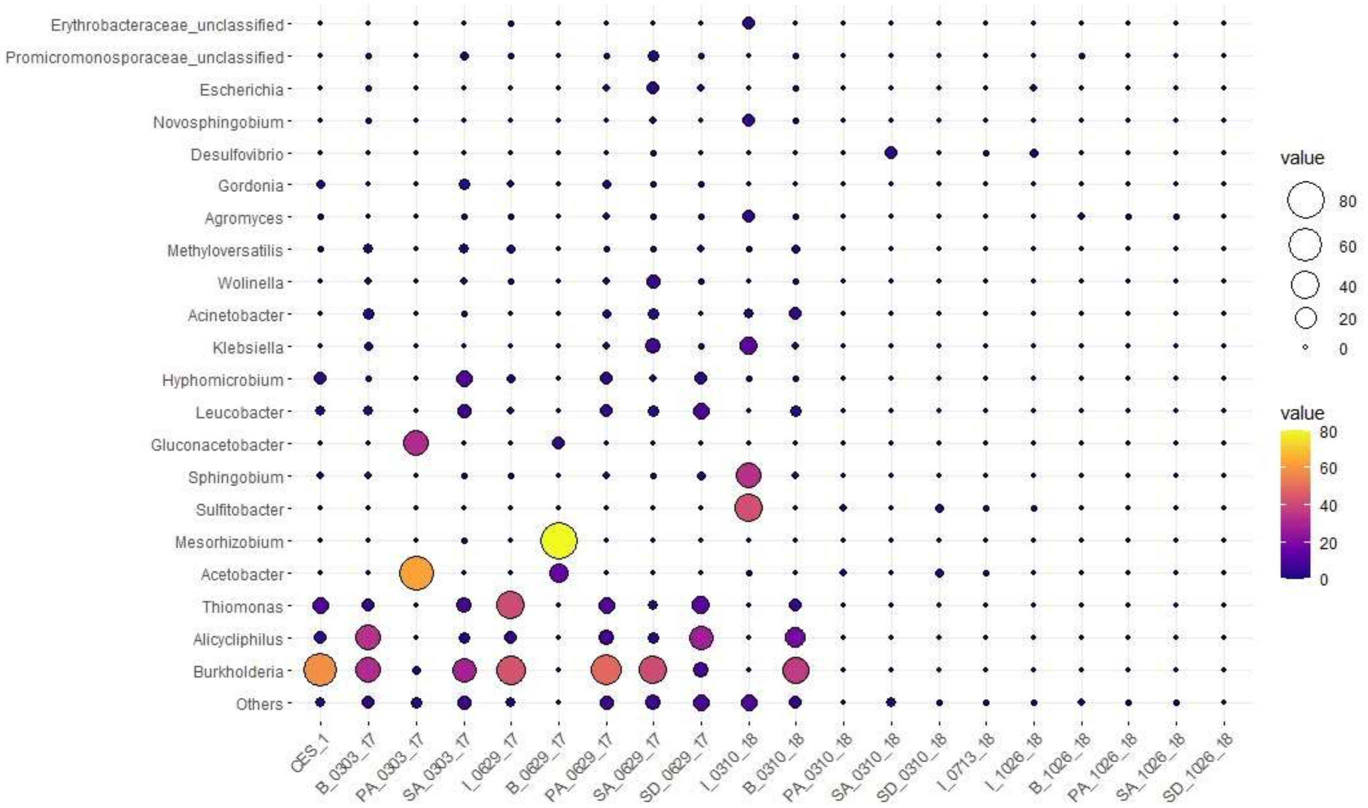

Figure S2. Changes in the abundance of the microbial communities over time at the genus level after bioaugmentation of the complex microbial consortium CES-1

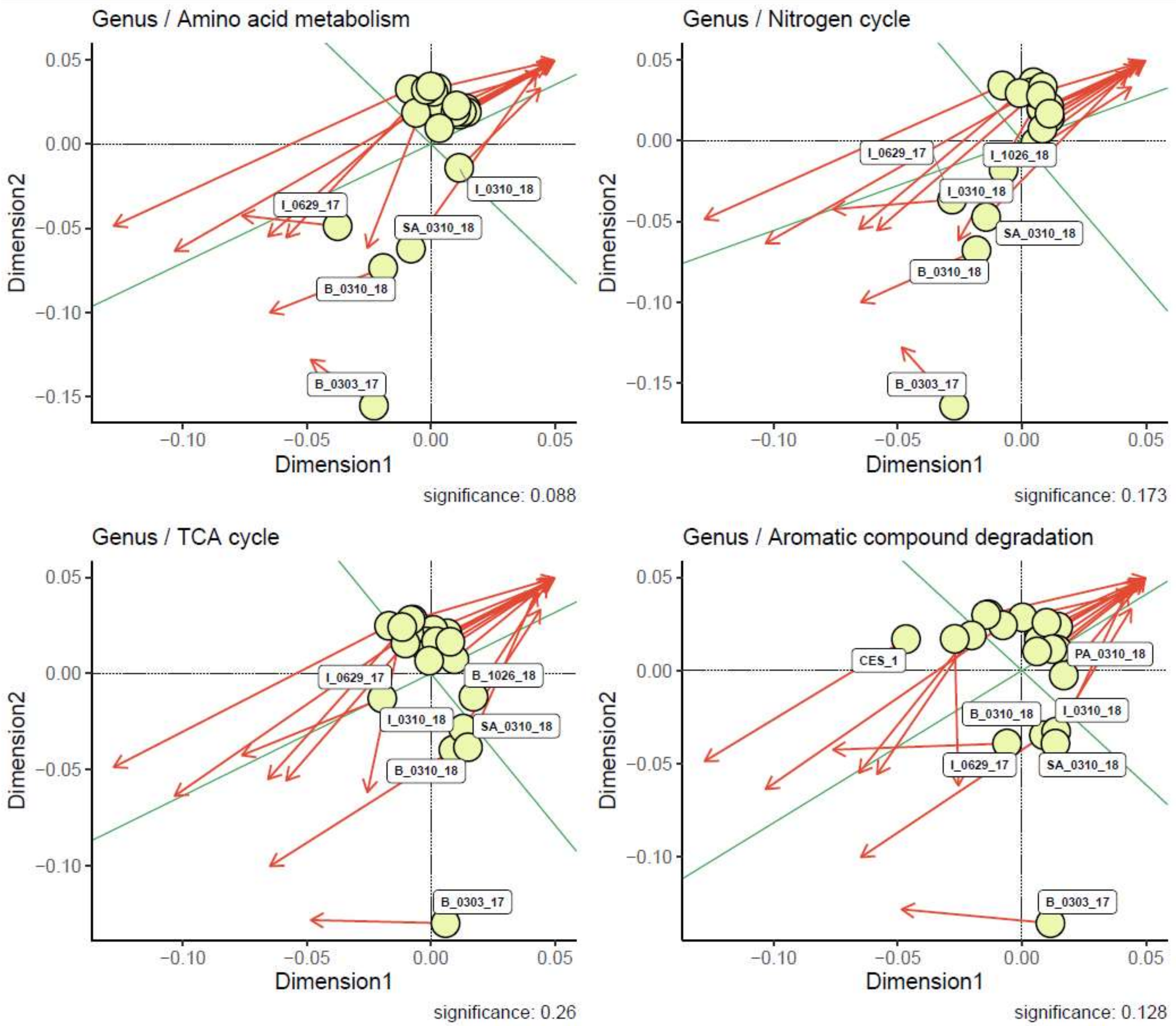

Figure S3. The Procrustes plot analysis of microbial community (Genus level) with different degradation pathways. The positive dimension is a nonnegative number, representing the credibility of the cluster results. Blue arrow indicates the significant positive correlation parameters (p<0.05).

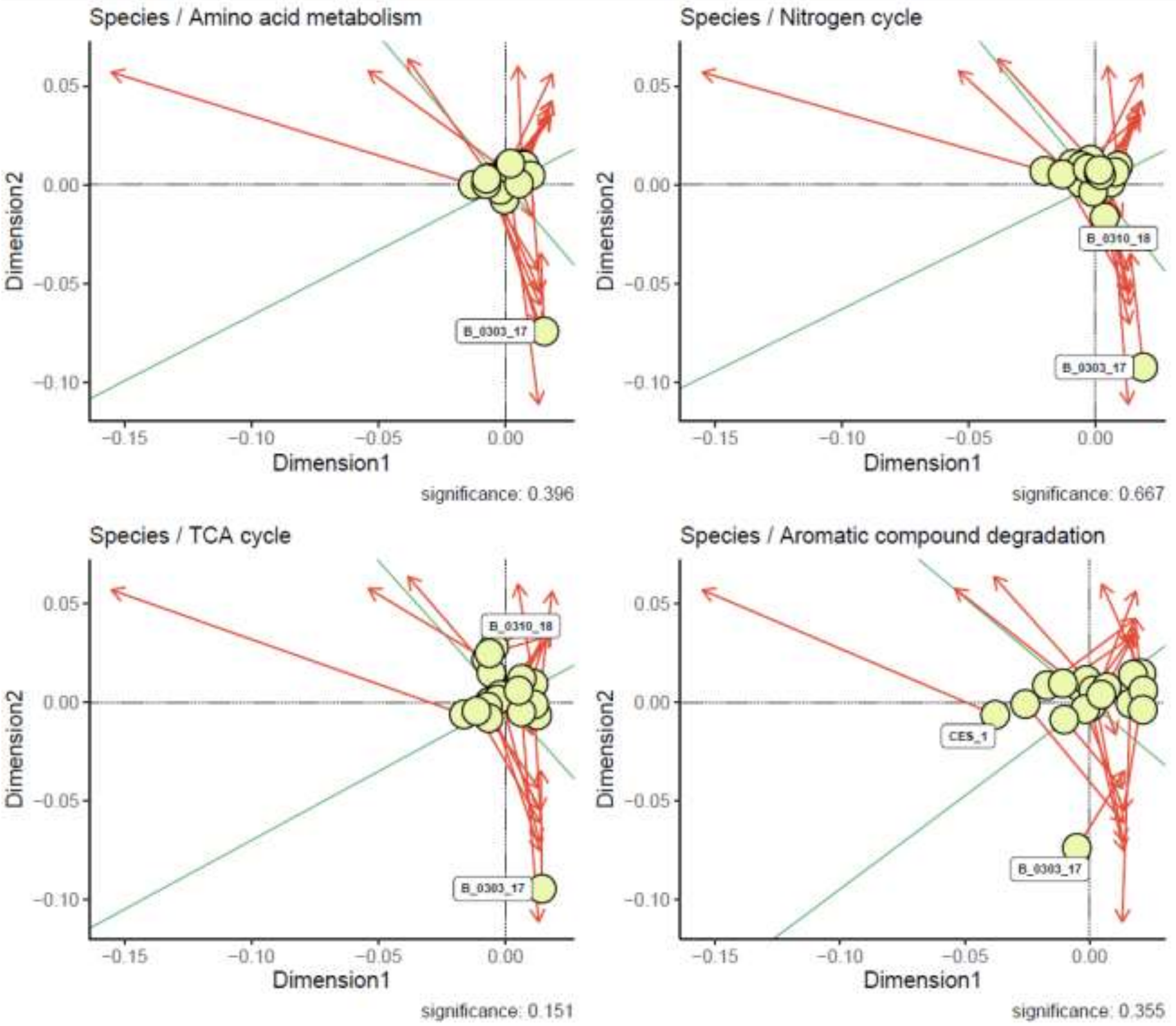

Figure S4. The Procrustes plot analysis of microbial community (species level) with different degradation pathways. The positive dimension is a nonnegative number, representing the credibility of the cluster results. Blue arrow indicates the significant positive correlation parameters (p<0.05).
